# Cell surface protein–protein interaction profiling for biological network analysis and novel target discovery

**DOI:** 10.1093/lifemedi/lnae031

**Published:** 2024-08-29

**Authors:** Jiaojiao Chen, Maoxin Fang, Yuwei Li, Haodong Ding, Xinyu Zhang, Xiaoyi Jiang, Jinlan Zhang, Chengcheng Zhang, Zhigang Lu, Min Luo

**Affiliations:** Institute of Pediatrics, Children’s Hospital of Fudan University, and Shanghai Key Laboratory of Medical Epigenetics, International Co-laboratory of Medical Epigenetics and Metabolism, Ministry of Science and Technology, Institutes of Biomedical Sciences, Fudan University, Shanghai 200032, China; Institute of Pediatrics, Children’s Hospital of Fudan University, and Shanghai Key Laboratory of Medical Epigenetics, International Co-laboratory of Medical Epigenetics and Metabolism, Ministry of Science and Technology, Institutes of Biomedical Sciences, Fudan University, Shanghai 200032, China; Institute of Pediatrics, Children’s Hospital of Fudan University, and Shanghai Key Laboratory of Medical Epigenetics, International Co-laboratory of Medical Epigenetics and Metabolism, Ministry of Science and Technology, Institutes of Biomedical Sciences, Fudan University, Shanghai 200032, China; Institute of Pediatrics, Children’s Hospital of Fudan University, and Shanghai Key Laboratory of Medical Epigenetics, International Co-laboratory of Medical Epigenetics and Metabolism, Ministry of Science and Technology, Institutes of Biomedical Sciences, Fudan University, Shanghai 200032, China; Institute of Pediatrics, Children’s Hospital of Fudan University, and Shanghai Key Laboratory of Medical Epigenetics, International Co-laboratory of Medical Epigenetics and Metabolism, Ministry of Science and Technology, Institutes of Biomedical Sciences, Fudan University, Shanghai 200032, China; Institute of Pediatrics, Children’s Hospital of Fudan University, and Shanghai Key Laboratory of Medical Epigenetics, International Co-laboratory of Medical Epigenetics and Metabolism, Ministry of Science and Technology, Institutes of Biomedical Sciences, Fudan University, Shanghai 200032, China; The Fifth People’s Hospital of Shanghai, Fudan University, Shanghai 200240, China; Department of Physiology, University of Texas Southwestern Medical Center, Dallas, TX 75390, USA; The Fifth People’s Hospital of Shanghai, Fudan University, Shanghai 200240, China; Shanghai Institute of Infectious Diseases and Biosecurity, Shanghai Medical College, Fudan University, Shanghai 200032, China; Institute of Pediatrics, Children’s Hospital of Fudan University, and Shanghai Key Laboratory of Medical Epigenetics, International Co-laboratory of Medical Epigenetics and Metabolism, Ministry of Science and Technology, Institutes of Biomedical Sciences, Fudan University, Shanghai 200032, China

**Keywords:** secretome protein–protein interaction, receptor–ligand interaction, deorphanization, high-throughput screening

## Abstract

The secretome is composed of cell surface membrane proteins and extracellular secreted proteins that are synthesized via secretory machinery, accounting for approximately one-third of human protein-encoding genes and playing central roles in cellular communication with the external environment. Secretome protein–protein interactions (SPPIs) mediate cell proliferation, apoptosis, and differentiation, as well as stimulus- or cell-specific responses that regulate a diverse range of biological processes. Aberrant SPPIs are associated with diseases including cancer, immune disorders, and illness caused by infectious pathogens. Identifying the receptor/ligand for a secretome protein or pathogen can be a challenging task, and many SPPIs remain obscure, with a large number of orphan receptors and ligands, as well as viruses with unknown host receptors, populating the SPPI network. In addition, proteins with known receptors/ligands may also interact with alternative uncharacterized partners and exert context-dependent effects. In the past few decades, multiple varied approaches have been developed to identify SPPIs, and these methods have broad applications in both basic and translational research. Here, we review and discuss the technologies for SPPI profiling and the application of these technologies in identifying novel targets for immunotherapy and anti-infectious agents.

## Introduction

The term secretome was first coined by Tjalsma et al. in 2000 to denote all the factors secreted by a cell [1]. It was later revised to include only proteins secreted into the extracellular space [1, 2]. Here, we define the secretome as all the secreted proteins and transmembrane proteins on the plasma membrane that are synthesized and transported via the secretory pathways [3, 4]. In the secretory pathway, signal sequence of proteins is usually required to allow them to be guided into the endoplasmic reticulum (ER) and then transported through the Golgi apparatus via vesicles, finally delivered to the cell surface [5, 6]. The signal sequence on secreted proteins is a short, hydrophobic N-terminal sequence called a signal peptide (SP) [5–7]. Membrane proteins may also contain the SP, but, in general, the N-terminal transmembrane (TM) region functions as the signal sequence [7, 8]. The human genome encodes ~2000 secreted proteins and ~5500 transmembrane proteins, accounting for ~36% of protein-encoding genes (www.proteinatlas.org, www.uniprot.org). Over half of the transmembrane proteins are UniProt annotated with cell membrane localization. However, the internal membrane-embedded proteins, particularly those associated with ER and Golgi apparatus, may reside on the cell surface due to the dynamic network formed among these membrane compartments, such as ERGIC3, FUT8, and MGAT2 [9–12]. The nuclear envelop (NE) and mitochondria show minimal connections with cell membrane, and NE is interconnected with ER, with many of the identified NE transmembrane proteins predominantly presenting in the peripheral ER [13–16]. Among the Uniprot-reviewed human transmembrane proteins, ~60 and 380 have specific annotations of NE and mitochondrial localization, respectively. Notably, some transmembrane proteins also have secreted isoforms [17]. For example, in addition to 4Ig- and 2Ig-membrane-embedded B7H3 isoforms, serum-soluble B7H3 has been reported in patients with carcinoma [18, 19]. ACE2, a coronavirus receptor, also has a soluble isoform [20] and has been recently reported to mediate SARS-CoV-2 entry via interaction with other transmembrane proteins [21]. The secretome includes all receptor and ligand proteins at the cell surface or in the extracellular space, and their interactions on the plasma membrane represent a major route of cell communication with the environment.

Secretome-based receptor–ligand interactions allow cells to receive or transmit signals that regulate a multitude of biological pathways required for cell survival, differentiation, proliferation, or to modulate pathogenic effects, and so on [22]. Extracellular ligands can be soluble, such as hormones, cytokines and growth factors, or membrane-associated, including transmembrane cell-adhesion molecules and adjacent cell surface proteins [2]. Dysregulation of ligand–receptor interactions can give rise to various diseases [23]. Extracellular recognition events also play critical roles in infectious diseases, as many pathogens use host–cell surface proteins for attachment, entry, and stimulation of diverse host-specific responses. SPPI analysis can be used to identify potential targets in life science research and drug development [24, 25]. Currently, 70% of FDA-approved drugs target secretome proteins [23, 24]. Despite the significance of SPPIs, a large number of orphan receptors and ligands exist, with many SPPIs underrepresented in current protein interaction datasets [23, 26, 27]. In addition, a receptor/ligand can have multiple functional partners and mediate different responses under different conditions [28–30].

Identifying the unknown receptor or ligand of a target protein is a challenging task. Most SPPIs are transient and/or weak, and the biophysical properties of membrane proteins impede screening at the genomic level [31, 32]. Conventional technologies, such as yeast two-hybrid technologies (Y2H), affinity purification mass spectrometry (AP-MS), mammalian protein interaction trap (MAPPIT) analysis, and luminescence-based mammalian interaction group (LUMIER) methods are not well-suited to the systemic detection of SPPIs [22, 23, 32–34]. Improvements in the receptor–ligand interaction screening technologies have resulted in more physiological-relevant and/or larger-scale screens, providing a set of powerful tools to fully map and understand SPPIs [23]. Here, we review and discuss the technologies used for SPPI profiling from aspects of principles, advantages, and disadvantages, and their application in identifying novel targets of anti-infectives and immunotherapy.

## Biophysical properties of secretome proteins

Numerous secretome proteins require various posttranslational modifications (PTMs) for functionality [35]. These modifications take place in the endomembrane system, including disulfide bond, glycosylation, methylation, acetylation, and phosphorylation [35–37]. The majority of these PTMs are absent in prokaryote cells, rendering prokaryotic expression systems ineffective for analyzing eukaryotic SPPIs [34, 38, 39]. Similarly, within the Y2H system, proteins are expressed in the reducing environment of the yeast cell and, therefore, may fail to acquire the native conformation existing in human cells [31, 33].

In addition, secretome proteins, particularly those bearing multiple transmembrane segments or a hydrophobic membrane-spanning region or a hydrophilic glycan coat, pose challenges in solubilization and biochemical manipulation [31], hindering the purification of correctly folded full-length proteins [23]. In addition, some secretome proteins only function upon cleavage [40]. For instance, the sonic Hedgehog (sHh) protein initiates a signaling cascade only when cleaved into two separate peptides [41, 42], and in G-protein-coupled receptors (GPCRs) with an autoproteolysis-inducing domain, appropriate proteolytic cleavage is required to become functional [40, 43], further complicating the isolation of functional proteins.

These properties notably hinder the application of high-throughput protein–protein interaction screening for secretome proteins. In addition, many SPPIs occur as transient interactions, characterized by low affinity (*K*_D_ in the µM–mM range) and very short half-lives (≤ 1 s) [23, 31, 32, 44]. Thus, a number of technologies have been developed in the past few decades to circumvent these obstacles while remaining high-throughput protein interaction capabilities.

## Secretome protein–protein interaction screening

Secretome protein–protein interaction screening approaches must solve two problems: overcoming the biophysical challenges of studying low-affinity extracellular transient interactions and achieving high-throughput detection. Transmembrane proteins account for ~77% of secretome proteins, serving as receptors or membrane ligands in SPPIs. The relative biochemical intractability of membrane proteins represents a major obstacle to SPPI screening. Therefore, based on the type of membrane protein library, current SPPI screening approaches can be classified into two broad categories, ectodomain (ECD)-based and cell-based (Table 1).

**Table 1.** Approaches for secretome protein–protein interaction screening

Current challenges to study SPPIs		(1)The amphipathic transmembrane proteins are difficult to solubilize and manipulate biochemically			(2)Transmembrane proteins require special conditions for correct folding and function		(3)Transient interactions, with low affinity and fast dissociation speed	
Approaches	Principles	Bait	Prey	Analysis	Advantages	Limitations	Technique names	Ref.
ECDs-based high-throughput screening	Use ECDs of transmembrane proteins to detect the interactions with other secretome recombinant proteins in the form of “bait”–“prey”.	Proteins, secreted polypeptides	Collection of purified recombinant proteins (secreted protein, soluble ECDs or binding domains of membrane proteins)	Chromogenic reaction or fluorescein	(1) The ECD of transmembrane receptors can retain their binding characteristics. (2) Multimerization strategies enhance the binding affinity and the sensitivity of detection for low-affinity interaction.	(1) Multi-pass membrane proteins and proteins that need transmembrane domains to be functional are intractable. (2) Preparation of ECD proteins is costly and needs complicated techniques.	Avidity-based extracellular interaction screen (AVEXIS), scalable arrayed multi-valent extracellular interaction screen (SAVEXIS), nucleic acid programmable arrays (NAPPAs)	[31, 32, 45–49]
	**Cell-based high-throughput screening**							
Screening based on chemical proteomic cross-linking reagents	Ligands coupling with chemical proteomic reagents are used to capture endogenously expressed receptors, and then the enriched binding complexes were analyzed by MS.	Proteins, peptides, viruses	Cells with endogenous receptor(s)	AP-MS	(1) Enable identification of multiple specific receptors for a given ligand under near-physiological conditions. (2) Allow the capture of weak and transient PPI within a few Å by covalent linkages.	(1) Rely on the endogenous expression of receptors. (2) Effects of oxidizing environment or UV irradiation on cell surface interactions need further verification.	Ligand–receptor capture technology (LRC) based on TRICEPS, ABS, and HATRIC; photo-cross-linking approaches	[50–56]
Screening based on a proximity labeling system	The labeling enzyme genetically fused with bait protein can label proximal proteins in the presence of substrates, then the labeled proteins can be isolated and analyzed by MS.	Proteins, viruses	Cells with endogenous receptor(s)	AP-MS	(1) Improve the low accuracy of traditional IP-MS in PPI detection. (2) Enable the detection of weak and transient protein–protein interactions with lower false-positive.	(1) Rely on the endogenous expression of receptors. (2) Unwanted self-modification of the enzyme is inevitable. (3) Labeled proteins may not be the directly binding partner.	Proximity labeling screening based on BioID, HRP, APEX, and PUP-IT system	[57–62]
Expression cloning	The cell clones transfected with specific cDNA will be screened by its ligand responsiveness.	Proteins, viruses	A cDNA library from the cells with endogenous receptor(s)	Usually gain of function	SPPIs can be studied within the cellular microenvironment and tissue context.	(1) The pools are derived from specific cell line or tissue. (2) Some weak PPIs would be lost after multiple subcloning steps. (3) If a complex (more than 2 proteins) is needed for interaction, this may not work.	Genome-wide gain-of-function screening	[63–68]
Extracellular interaction screening using CRISPR technology	A genome-scale sgRNA library is transduced into cells, then target gene can be screened out by binding or specific phenotypes of cells.	Proteins, viruses	Cells with endogenous receptor(s)	Usually loss of function	(1) The SPPIs can be studied within the cellular microenvironment and tissue context. (2) Identify not only receptors, but also regulators involved in PPIs and the readout is phenotype driven.	(1) CRISPR-KO screening is to some extent limited by the cell lines or functional models used. (2) CRISPRa screening is limited by the receptor expression on cell surface.	CRISPRa enrichment screening; CRISPR-KO screening	[69–74]
Genome-scale secretome interaction arrays	A cDNA library is individually transfected into cells and the target gene is screened out by ligand-binding capacity or specific phenotype.	Proteins, viruses	Collection of secretome coding genes	Fluorescence detection, or gain of function	(1) The assay was not limited by cell lines or membrane protein types. (2) A complete interaction mapping with a specific probe can be observed in a single round of screening.	(1) Rely on the cell’s ability to overexpress and transport receptors to the cell surface. (2) Transfection of individual receptor plasmid will limit the PPIs’ detection of multi-subunit complexes.	Genome-scale T-cell activity array (TCAA); genomic receptor profiling	[12, 75–79]

**Figure 1.**
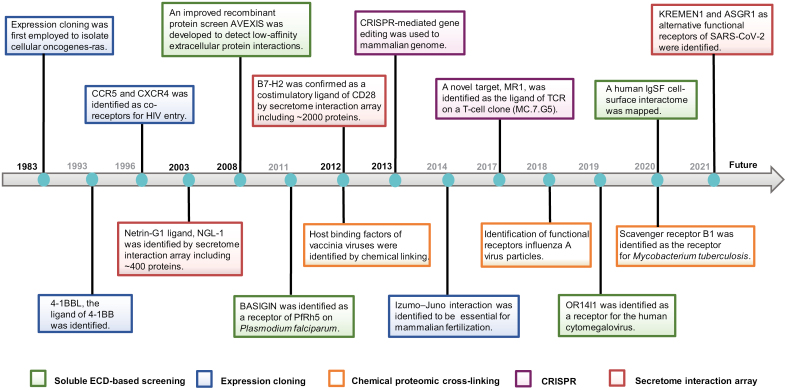
**Milestones in secretome protein–protein interaction screening studies.**Representative discoveries utilizing cell-based or ECD-based high-throughput screening approaches.

ECD-based high-throughput screening utilizes the recombinant soluble ECDs of transmembrane proteins as a library. The ECDs of membrane receptors can be expressed in eukaryote cells and purified in soluble form. While cell-based high-throughput screening utilizes the endogenous receptor repertoire in a specific type of cells as an existing library, or ectopically expressing a collection of membrane proteins on mammalian cells. It overcomes the challenges such as solubility, folding, and covalent modifications, and enables cell surface interactions to be studied within a more physiologically relevant environment. The milestones of SPPI screening technology are illustrated in Fig. 1.

### Ectodomain-based high-throughput screening

**Figure 2.**
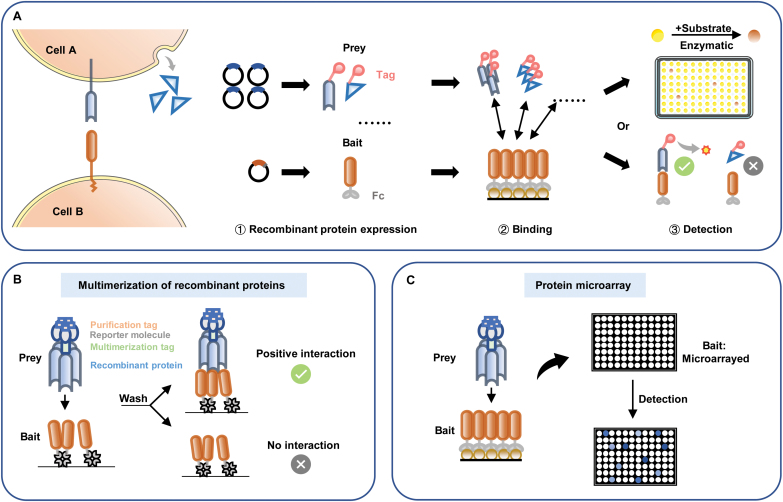
**Scheme showing the high-throughput screening based on the soluble ECD of transmembrane proteins.**(A) A summary of the assay is shown. The “bait” fused with a tag is immobilized on the solid surface via protein A binding. A “prey” recombinant protein is expressed as a tag- or enzyme-fused protein. Binding of “bait” and “prey” can be detected by fluorescent signals or chromogenic reactions of the substrate. (B) Prey and bait are multimerized via their corresponding tag so that low-affinity PPIs can be detected. (C) In a protein microarray assay, thousands of bait proteins are immobilized on a slide, and protein–protein interactions are detected via fluorescence signals.

ECD-based approaches rely on the ability to express the ECDs of membrane proteins as secreted recombinant proteins that retain the extracellular binding activity [31, 32]. The recombinant proteins are usually expressed in mammalian or insect cell lines to obtain the appropriate PTM for proper folding and function. Using ECDs simultaneously overcomes the difficulties associated with insoluble hydrophobic transmembrane regions and enables the addition of protein tags for facile manipulation and detection. ECD-based methods, like ELISA, use a “bait and prey” approach, with one immobilized on a solid surface while another contains a reporter in solution for binding measurement [80]. The reporter is usually β-lactamase or human placental alkaline phosphatase fused to the recombinant proteins, enabling detection by chromogenic reaction of the substrate [45, 46]. It can also be a fluorescein that coupled directly to the protein [47] (Fig. 2A).

To increase the binding affinity of transient interactions and sensitivity of the assay, recombinant proteins are expressed as multimers by adding a human IgG Fc domain or the pentamerization domain of a rat cartilage oligomer matrix protein [32, 44–46] (Fig. 2B). The avidity-based extracellular interaction screen (AVEXIS) was specifically developed to detect low-affinity extracellular protein interactions [46]. In this method, pentamerization can increase the affinity of prey proteins and improve detection sensitivity by at least 250-fold. This technology can detect very transient interactions (half-lives ≤ 0.1 s) with a low false-positive rate. A number of receptor–ligand interactions have been identified using this approach, such as those for merozoite invasion of erythrocytes [81], zebrafish early development [82], and neural guidance and interconnectivity [83, 84], and for HCMV infection in various cells [44]. The approach has also been used to reveal the cell surface interaction networks for extracellular IgSF, FnIII, and LRR protein families in *Drosophila melanogaster* [45], create an interaction profile of neural leucine-rich repeat receptors [85], characterize the interactions on the surface of human leukocytes [48], and map a human IgSF interactome [26]. Although the sensitivity is substantially increased, AVEXIS is not suitable for detecting homophilic extracellular interactions, as prey–prey associations with high affinities prevent prey–bait interactions and increase the false-negative rate [46].

Protein microarray technologies can expand the scale of sample analysis and reduce the sample quantities required for each interaction assay to achieve high-throughput screening [86]. In a microarray, thousands of proteins are immobilized on a slide, and protein–protein interactions (PPIs) are detected via fluorescence signals [87–89] (Fig. 2C). In one such large-scale microarray screen, the E3 protein of human adenovirus was tested for potential interactions with 1500 human transmembrane proteins, and 51 previously unknown virus–host interactions were discovered and validated [88]. However, the long process of printing recombinant proteins onto the slides may compromise the binding function of proteins [23]. An alternative microarray format, nucleic acid programmable arrays (NAPPAs), can directly perform complementary DNA (cDNA) transcription and translation *in situ* on slides [49, 90], but these proteins will not have PTMs, which may affect receptor binding properties.

These technologies are suitable for polypeptides, secreted protein, and soluble ECDs, as well as the binding domains of membrane proteins that remain functional when expressed. Multi-pass membrane proteins (such as GPCRs) and proteins that need multiple subunits to function cannot be analyzed using this assay. The preparation of a large recombinant protein library requires sophisticated, technically challenging, and expensive methodologies, which may be beyond the scope of many laboratories, limiting the application of these assays.

### Cell-based high-throughput screening

**Figure 3.**
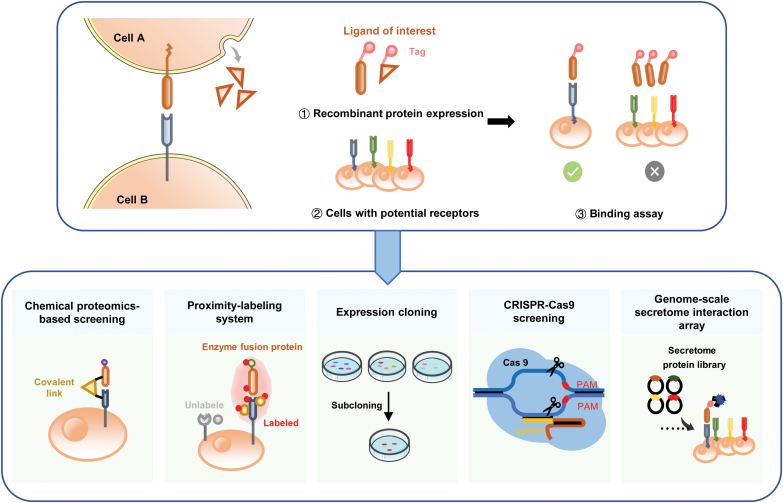
**Scheme depicting cell-based high-throughput screening.**Cell-based screening can be used to analyze the extracellular interactions of full-length membrane proteins on the cell surface. We divide the approach into five different assays: screening based on chemical reagents, proximity-labeling system, expression cloning, CRISPR-Cas9, and genome-scale secretome arrays.

Cell-based high-throughput screening is used to investigate extracellular interactions with full-length membrane proteins being expressed on the cell surface, making it possible to detect the interaction of a given ligand to the receptor with either single-transmembrane or multiple transmembrane domains (Fig. 3).

#### Screening based on chemical proteomic cross-linking reagents

Mass spectrometry (MS) can be used to identify PPIs in cell lysates. However, the transient nature of SPPIs, their hydrophobicity, and the low abundance of plasma membrane proteins present challenges for unbiased and accurate mapping of receptor–ligand interactions using MS [51]. To solve these problems, chemical proteomic cross-linking strategies have been developed, allowing the capture of weak and transient PPIs under native conditions via covalent linkages [51–53].

**Figure 4.**
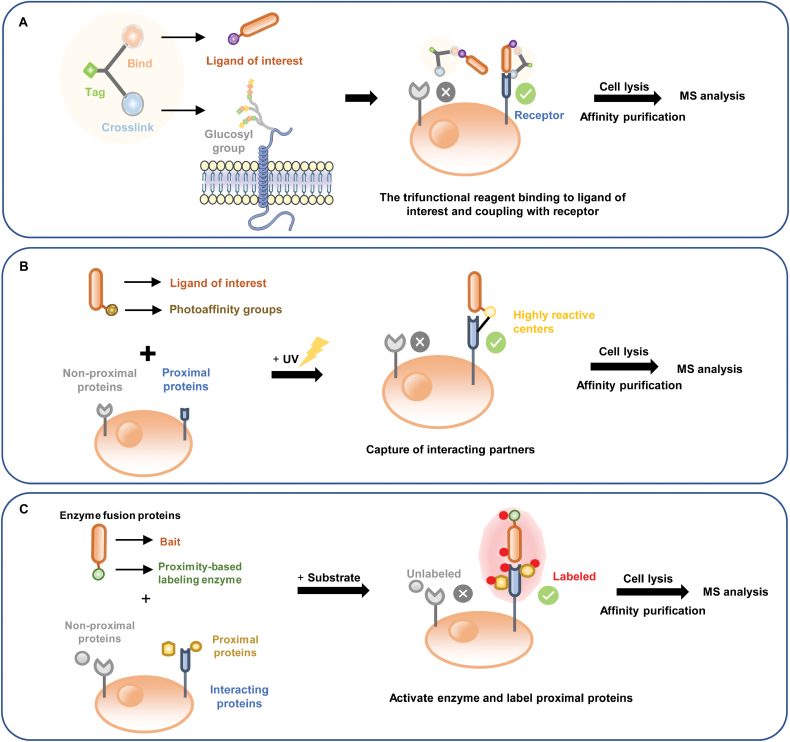
**Schemes illustrating screening assays based on chemical proteomics and proximity labeling.**(A) The assay depends on MS and a trifunctional cross-linking reagent. The reagent binds ligands of interest and crosslinks with glycosylated receptors on living cells. It also has a tag that enables affinity purification and subsequent quantitative MS. (B) The assay depends on a photoaffinity group that is converted into a highly reactive center upon UV exposure, which rapidly inserts into a neighboring X–H bond, converting noncovalent interactions into covalent interactions. After subsequent treatment, high mass-accuracy MS is performed for further analysis in both assays. (C) Genetic manipulation is used to fuse a bait protein to a proximity-based labeling enzyme. In the presence of substrate, the fused enzymes generate reactive radicals to covalently tag the neighboring proteins. The tagged proteins can be isolated by affinity capture for further MS analysis.

The trifunctional cross-linking reagents (such as TRICEPS, ASB, and HATRIC) are designed such that one group binds the ligand via an amino group, and a second group crosslinks with aldehydes on the glycosylated receptor, and there is a tag that enables affinity purification and subsequent quantitative MS [50, 51, 54, 55]. In this system, cells are treated with oxidant to produce the receptor-linked aldehydes, and the ligands of interest, labeled with crossing-linker probes, are then added. Labeled ligands are captured via the purification tag, and cross-linked binding proteins are enriched for MS analysis (Fig. 4A). Ligand–receptor capture technology (LRC) based on TRICEPS, ABS, and HATRIC enables unbiased and sensitive identification of multiple specific receptors for secretory proteins, polypeptides, therapeutic antibodies, and virus particles [51, 91]. This technology does not require any genetic manipulation; however, only glycosylated receptors can be identified.

Photo-cross-linking approaches utilize unnatural amino acids (UAAs) to study PPIs in living cells. UAAs incorporating photo-cross-linkers are site-specifically incorporated into the “bait” proteins via expanded genetic-code technologies. When irradiated with UV light, the activated cross-linker covalently captures nearby organic molecules, including interacting partners, which can then be affinity purified and analyzed by immunoblotting or MS [52, 53, 56, 92–94] (Fig. 4B). Photo-crosslinker approaches have been widely applied to study a range of PPIs and their interaction interfaces, including chaperone–substrate interactions [95, 96], ligand–receptor interactions [97–99], membrane protein–membrane protein interactions [100, 101], and so on. This technology requires genetic manipulation, and nonspecific contamination and false-negative identification need to be minimized or eliminated.

Chemical cross-linker-based screening can capture transient interactions within a few Å of the bait under near-physiological conditions [52]. However, the effects of oxidizing environment or UV irradiation on SPPIs detection need to be investigated further.

#### Screening based on a proximity labeling system

Proximity labeling systems have been established to identify PPIs in naïve cellular environments, including those based on BioID (proximity dependent biotin identification), HRP (horseradish peroxidase), APEX (engineered ascorbate peroxidase), and PUP-IT (pupylation-based interaction tagging) system [57–60]. The technology is based on the genetic fusion of a bait protein with a proximity-based labeling enzyme, such as biotin ligase or peroxidase, which can label proteins neighboring the bait protein in the presence of substrates. The labeled proteins can then be isolated by affinity capture and analyzed by MS [58, 61, 62] (Fig. 4C). This approach has been used to study host–pathogen interactions [102–109].

Proximity labeling systems greatly improve the accuracy of traditional IP-MS in PPI detection and enable the detection of weak and transient PPIs with lower false-positive rates. However, most enzymes used in proximity labeling undergo unwanted self-modification, potentially inactivating the enzyme, depleting substrate, and introducing background signals into subsequent analyses. The labeling radius of proximity labeling is estimated to be ~10 nm [59, 60], so these types of approaches will label proteins that bind directly or indirectly to the bait protein.

The proximity biotinylation strategy has been adapted for mouse models, enabling *in vivo* labeling, detection, and enrichment of secretome proteins in a tissue/cell-type specific manner [110–113]. A floxed transgenic mouse expressing ER-BioID^HA^, termed the “secretome mouse,” has been generated, allowing rapid identification of the secretome from any cell or tissue under basal conditions or following a physiological or pathophysiological stress [114].

#### Expression cloning

**Figure 5.**
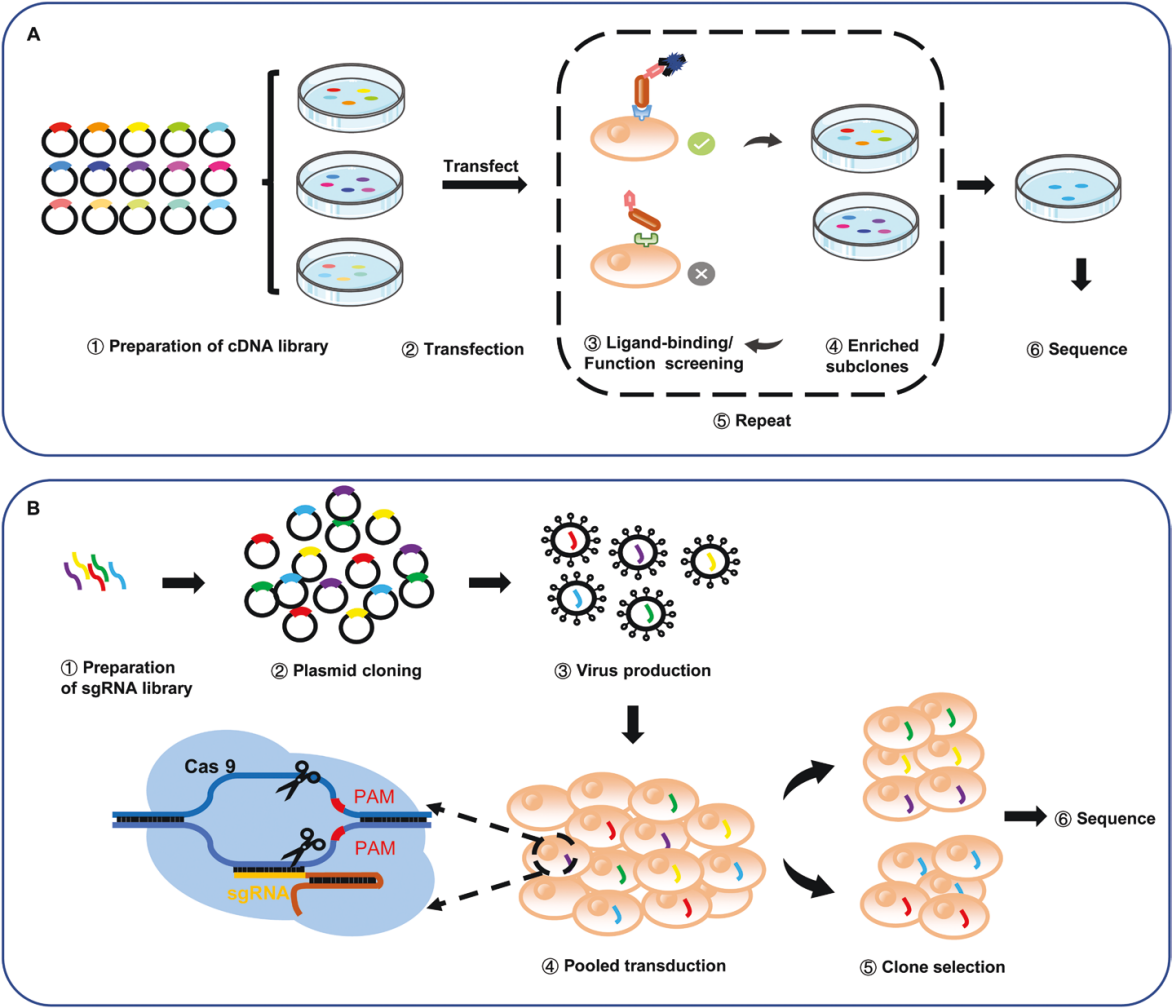
**Schemes showing expression cloning and CRISPR–Cas9 screening assays.**(A) The cDNA library is transfected into cultured cells and screened on the basis of either ligand binding or the ligand-induced response. Enriched plasmids are reintroduced into a cultured cell line for further enrichment until the receptor clone is selected and identified by sequencing. (B) Cells are transduced with a lentivirus library containing sgRNAs targeting the entire/selected protein-coding gene repertoire in the genome. After fluorescence-activated cell sorting or antibiotic selection, the sorted populations are subjected to next-generation sequencing to determine the frequency of each sgRNA. Comparing sorted and control populations, genetic factors of interest can be identified.

Expression cloning technologies were initially widely used to discover the known receptor of a ligand (or virus). In general, a specific cell type that is responsive to ligand stimulation is required to construct a complementary DNA (cDNA) library, which is then transfected into a nonresponsive cell line to identify the cDNA that confers ligand responsiveness [63, 115] (Fig. 5A).

Multiple rounds of subcloning are often required to obtain cell clones with superior binding or responsiveness to the ligand [63]. Many viral receptors [64–66, 116, 117] and growth factor receptors [118–120] have been identified using this method. Adapted versions of the technology have been used to study the low-affinity binding of sperm and egg during fertilization [121]. TMEM120A was identified as a host factor that regulates Zika virus infection, using an adapted version of the technology, in combination with a genome-wide cDNA library [68]. Major drawbacks of expression cloning technologies include that the receptor(s) are limited to a specific cell type or tissue, and some weak PPIs may be lost after multiple rounds of subcloning [67].

#### Extracellular interaction screening using CRISPR technology

The clustered regularly interspaced short palindromic repeats (CRISPR)/Cas9 system provides a powerful gene-editing technology, allowing facile genome-scale loss-of-function and gain-of-function gene analyses in living cells [70, 122–124]. The technology has been used extensively to study gene interaction networks underlying biological processes such as signal transduction and host–pathogen recognition [71, 72, 125] (Fig. 5B).

Multiple genome-scale CRISPR/Cas9 genetic screening platforms have been developed for cell surface receptor identification [73, 126–130]. The method involves transduction of a lentivirus-containing sgRNA library into cells, and screening for: (i) a loss-of-binding phenotype; (ii) a change in downstream reporter signals; or (iii) the cells becoming refractory to pathogen infection. In addition to proteins and antibodies, pathogens (such as virions and bacteria) and small molecules can also be used as the ligand to screen their relevant receptors on the cell surface. With this approach, host factors involved in the entry of dengue virus, hepatitis C virus, murine norovirus, SARS-CoV-2, and other viruses have been identified [72, 126, 131–135].

The ability to use the CRISPR/Cas9 system for transcriptional activation of endogenous genes (CRISPRa) has made gain-of-function screening possible [69, 70]. A nuclease-inactive mutant Cas9 protein is fused with a transcriptional activator domain and recruited to the promoter region of the target gene through the sgRNA, leading to the overexpression of the target gene in mammalian cells [74]. In contrast to CRISPR-KO screening, CRISPRa screening does not require prior identification of a cell line that can bind or respond to target ligands. CRISPRa screening has identified a novel set of receptor–ligand interactions, providing an effective approach to discovering the novel interacting partners for orphan ligands/receptors including seven transmembrane G-protein-coupled receptors [74, 136].

Compared to other PPI screening systems, CRISPR-based screening can identify not only the binding proteins that interact directly with “bait” proteins but also the factors that regulate the expression, processing, and transportation of these binding proteins [137]. For example, several regulatory molecules for checkpoint PD-L1 expression on the plasma membrane [138, 139], and the chaperone for the expression of BSG, an erythrocyte receptor for *P. falciparum*, were discovered using this type of approach [140]. Thus, candidate proteins obtained by CRISPR-based screening are not restricted to direct PPIs, which needs further determination.

#### Genome-scale secretome interaction arrays

**Figure 6.**
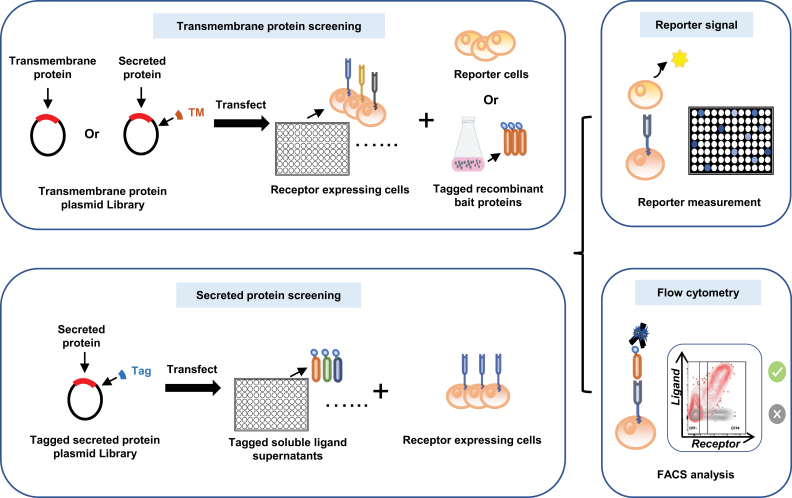
**Scheme illustrating genome-scale secretome interaction array approaches to screen transmembrane proteins (upper) or secreted proteins (down).**For the former, plasmids encoding transmembrane proteins or secreted proteins engineered to contain a transmembrane domain are transfected into mammalian cells individually, followed by incubation with the tagged recombinant bait proteins or reporter cells. For the latter, plasmids encoding tagged secreted proteins are transfected into mammalian cells individually, and ligand supernatants are collected for binding with the receptor-expressing cells. Receptor–ligand pair interactions are determined by the reporter activity or the tag labeling and detection with high-throughput screening flow cytometry.

Genome-scale secretome interaction array enables individual testing of each secretome protein under physiological conditions. A cDNA library is required for secretome interaction arrays, similar to expression cloning approaches. However, the former is a designed collection of individual cDNA clones of secretome proteins, while the latter is in a form of mixture, encoding both secretome and intracellular proteins of a specific cell type or tissue. In secretome interaction arrays, the cDNA clones are transfected individually into mammalian cells in multi-well plates for SPPI screening, either by functional assays or binding assays. Functional readouts can include response to ligand stimulation, induction/inhibition of a reporter, or viral infection. This type of readout enables the identification of phenotype-associated receptor(s) or ligand(s). For example, Siglec-15, a membrane protein, was identified as a novel T-cell inhibitory regulator using a genome-scale T-cell activity array (TCAA), in which an engineered T-cell served as a reporter [79]. However, the receptor for Siglec-15 on T-cells remains unknown, illustrating that if a whole cell or virus is introduced as a reporter or stimulus, the exact receptor(s) or stimulating molecule(s) still needs further investigation. For binding readouts, the transfected cells are incubated with the tagged recombinant bait protein, and the tags, usually mouse IgG2a Fc, human IgG1 Fc, or FLAG, can be labeled with fluorescent conjugated antibodies for measurement using high-throughput flow cytometry or fluorescence microscopy [12, 141] (Fig. 6).

Using these types of platforms, many functional SPPIs associated with immunity have been identified and/or validated, such as B7-H2/CD28 [76], B7-H5/CD28H [77], HVEM/SALM5 [142], SEMA4A/ILT-4 [143], and FGL1/Lag-3 [144]. Genomic receptor profiling performed with the spike protein of SARS-CoV-2 as the target, using a library containing >90% (5054) of annotated human transmembrane protein-encoding genes, revealed a host receptome of SARS-CoV-2 and identified Kremen1/ASGR1 as two alternative receptors that mediate ACE2-independent virus entry [12]. This technology could also be used to screen soluble ligands for a specific cell surface receptor: A tag is fused to all secreted proteins, and the supernatants containing these proteins are individually incubated with target receptor-expressing cells for subsequent labeling and detection (Fig. 6). To achieve higher-throughput detection, microarray technology can be applied to these methods: Nanolitre volumes of plasmid DNA are printed on a slide for reverse transfection to create a microarray with features consisting of clusters of transfected cells, providing a convenient means for SPPI screening on a large scale [145–147].

Overall, genome-scale secretome interaction arrays enable unbiased profiling of SPPI networks under physiological conditions, with the candidates being obtained by a single round of screening and not limited to specific cell types or membrane protein types. The major technical limitation of these approaches is that proteins with low cell surface expression are hard to detect.

## Secretome protein interaction screening applications

**Figure 7.**
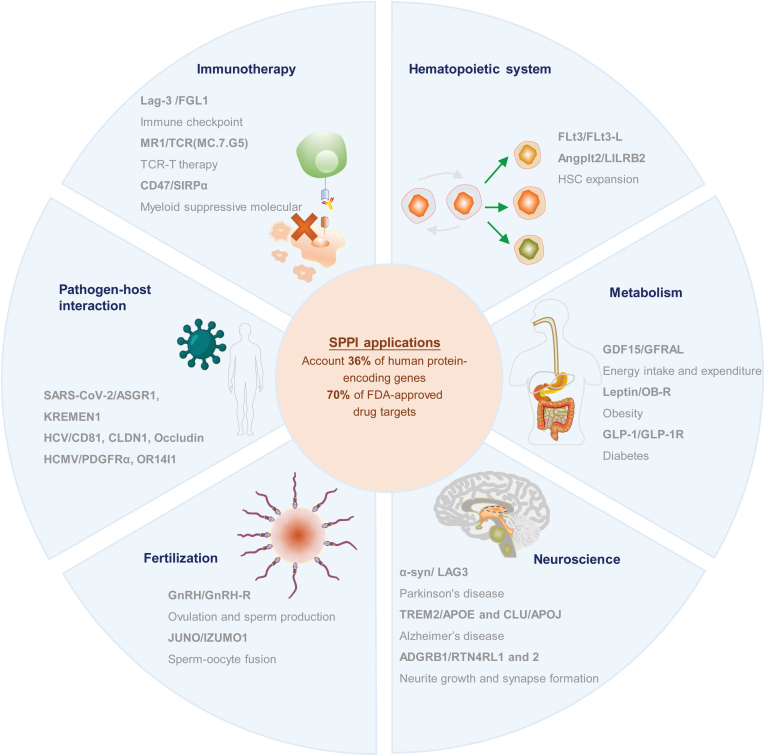
SPPI screening applications in multiple fields.

SPPI screening has a wide range of applications in both basic and translational research, as well as in the pharmaceutical industry. Progress in high-throughput SPPI screening has led to numerous ligand–receptor interaction discoveries in multiple fields, including immunotherapy [144, 148–150], hematopoietic research [151, 152], metabolism-related diseases [153–155], neuroscience [156–159], fertility [121, 160–162], and pathogen–host interactions [65, 144, 163–168] (Fig. 7). Here, we review pathogen–host interactions and immunotherapy to illustrate various applications of SPPI screening.

### Identification of pathogen–host interactions

A wide range of pathogens, including viruses, bacteria, fungi, and parasites, threaten human health. In 2020, 1044 new virus species were added to the official list of the International Taxonomy of Viruses (ICTV) (ictv.global), with thousands more waiting to be described and named [169]. The interaction between proteins on a pathogen’s surface and host cellular receptors is the first and most critical step in infection and pathogenesis [170]. Recent advances in SPPI screening have increased our ability to analyze a broad range of host–pathogen interactions (Table 2), facilitating the development of novel therapeutics and vaccines.

**Table 2.** Examples of pathogen–host interactions identified by SPPI screening approaches.

Screening approaches	Pathogen	Host factor	Ref.
ECD-based high-throughput screening	*Plasmodium falciparum*	Basigin (CD147), semaphorin-7A (CD108), integrin αvβ3	[81, 171, 172]
	HAdVs	SLAMF3/4/5/6/7/8, CD45, LILRB1/2, CD300A/C, EPHA3/6	[88]
	HCMV	Npr2	[44]
	*Streptococcus*	Fibronectin, fibrinogen, and C4BP	[173]
Chemical proteomic cross-linking reagents	*Vaccinia viruses*	AXL, M6PR, DAG1, CSPG4, and CDH13	[51]
	IAV	24 virus-interacting candidates	[55]
Expression cloning	HIV	CCR5, CXCR4, human mannose receptor (hMR)	[65, 165, 166]
	HCV	CD81, CLDN1, Occludin (OCLN)	[64, 116, 117]
CRISPR technology	Arthritogenic alphaviruses	Mxra8	[174, 175]
	HCMV	PDGFRα, OR14I1	[127, 128]
	Murine norovirus	CD300lf, CD300ld	[126, 129]
	ZIKV	AXL, αvβ5, TMEM120A	[68, 176, 177]
	VEEV	LDLRAD3	[130]
	Bat influenza virus	MHC-II, HLA-DR	[178]
Genome-scale secretome interaction arrays	SARS-Cov-2	ACE2, ASGR1, or KREMEN1	[12]
	SARS-Cov-2	DC-SIGN, L-SIGN, LSECtin, ASGR1, CLEC10A	[78]
	*Plasmodium falciparum*	EPCR	[147]
	Hom-1 calicivirus	hJAM1	[179]

ECD-based high-throughput screening systems have been used to identify and/or confirm the receptors used by many pathogens, providing targets for prevention and treatment. Severe and/or fatal malaria is predominantly caused the *Plasmodium falciparum* parasite. Using the AVEXIS assay, in combination with an erythrocyte membrane protein-derived recombinant ECD library, PfRh5/BASIGIN, MTRAP/Semaphorin-7A (CD108), TRAP/human integrin αvβ3 interactions were identified, and disruption of these interactions blocked malaria parasite entry into human tissues [81, 171, 172]. Similarly, Npr2 was identified as a receptor of human cytomegalovirus (HCMV) in endothelial cells and epithelial cells and neutralizing antibodies that block binding to Nrp2 effectively inhibited HCMV invasion [44]. Using three known human ligands (fibronectin, C4BP, and fibrinogen) as probes, Fib on the surface of *Streptococcus pyogenes* and *Streptococcus lactis* was identified as a protein required for invasion by protein microarray [173].

Cell-based high-throughput screening allows the detection of host–pathogen interactions under a relative physiological condition. Using TRICEPS to label cowpox viruses, AXL, M6PR, DAG1, CSPG4, and CDH13 were identified as receptors on human cells [51]. HATRIC-based ligand receptor capture (HATRIC-LRC) has been used to identify host receptors for influenza A virus (IAV) [55].

Expression cloning initiated the era of viral receptor discovery in the 1990s. CCR5 and CXCR4 were identified as co-receptors for HIV-1 entry into CD4^+^ T-cells [65, 165], and human mannose receptor (hMR) was found to regulate CD4 independent HIV-1 infection of astrocytes, contributing significantly to HIV-1 induced neuropathogenesis [166]. Chronic hepatitis C virus (HCV) infection is a leading cause of liver disease. Through expression cloning, researchers discovered the CD81 receptor as a key factor for HCV entry via a direct interaction with E2 protein [64] and unveiled claudin-1 (CLDN1) and occludin (OCLN) as the essential co-receptors [116, 117]. Transgenic mice expressing human CD81 and CLDN1 receptors permit persistent HCV infection, thereby offering an effective mouse model for chronic hepatitis C mechanistic studies and therapeutic strategy development [180].

CRISPR/Cas9 knockout-based screening has significantly facilitated the identification of host receptors for a wide array of pathogens [174, 175, 181]. Mxra8 was identified as an entry receptor for multiple emerging arthritogenic alphaviruses [174]. Subsequent studies showed that the Mxra8–E2 interaction was able to initiate pathological processes [175, 181]. With a genome-wide CRISPR screening, PDGF receptor-α (PDGFRα) was identified as a host receptor required for infection of HCMV viroid containing only trimeric complexes (trimeric viruses only) [128]. Similarly, the multi-transmembrane protein OR14I1 was identified as a receptor for HCMV [128]. Using a genome-wide sgRNA library, murine CD300lf and CD300ld were identified as functional receptors for murine norovirus (MNoV) [129], and CD300lf expression in human cells was found to break the species barrier that limits MNoV replication to mice [126]. CRISPR/Cas9 knockout-based screening also contributed to identifying host–cell receptors for ZIKV [68, 176, 177], VEEV [130], Bat influenza virus [178], and a range of other pathogens [130, 131, 182].

COVID-19 poses a significant threat to human health. The ACE2 receptor alone cannot explain the clinical differences between SARS-CoV-2 and SARS-CoV. Genome-scale secretome interaction array identified a panel of human membrane proteins that bind the viral S-protein. In addition to ACE2, ASGR1 or KREMEN1 can mediate SARS-CoV-2 infection but not SARS-CoV infection *in vitro* and *in vivo*. A cocktail of neutralizing antibodies blocking all three entry receptors had a synergistic effect in cell lines and human lung organoids [12]. Using a myeloid cell-related receptor array (consisting of ∼300 host membrane proteins), several S-protein binding receptors were discovered, which may mediate the proinflammatory responses that correlate with COVID-19 severity [78]. LRRC15 was also identified from a cell array including 2363 full-length human cell surface membrane proteins, it can interact with the SARS-CoV-2 spike protein and modulate the host cell’s susceptibility to the virus [183]. DC8-PfEMP1 on the surface of *P. falciparum* is critical for parasite invasion. Endothelial protein C receptor (EPCR) was identified as a receptor for DC8-PfEMP1 using Retrogenix cell microarrays against 2505 full-length secretome proteins, which may lead to the development of adjuvant therapies and vaccines [147]. Similarly, a Hom-1 cupulovirus-interacting receptor, human junctional adhesion molecule 1 (hJAM1), has also been identified [179].

SPPI profiling provides a powerful tool for understanding the mechanism of pathogenesis for a wide range of pathogens, as well as identifying targets for vaccines and drug development. Currently, a variety of related drugs and treatments based on SPPI profiling have been approved by the FDA or in clinical trials, such as *P. falciparum* (NCT04318002; NCT00890019), HIV (Maraviroc, FDA approved; leronlimad, NCT03902522II; Calimmune, NCT01734850, SB-728-T NCT01252641).

### Applications in immunotherapy

An imbalance in immune homeostasis underlies a range of diseases [184], with a number of SPPIs playing critical roles in the initiation of processes such as the inflammatory cascade and tumor immune escape [185, 186], and proteins involved in these interactions are emerging as attractive therapeutic targets [187]. Drugs blocking CTLA-4 and PD-1/PD-L1 inhibitory checkpoints have demonstrated durable clinical effects in a subset of cancer patients. The discovery of new immune inhibitory and stimulatory pathways will assist in designing novel strategies for overcoming immune tolerance and assisting antitumor responses.

VISTA (B7-H5) is an inhibitory receptor expressed on naive T-lymphocytes. Using ECD-based high-throughput screening approaches, VSIG-3 [188] and PSGL-1 [189] were identified as functional ligands for VISTA. Both interactions were shown to inhibit human T-cell proliferation and cytokine production [190], and clinical trials of VISTA-targeted cancer therapies are underway. Recently, Human leukocyte antigen (HLA)-E and HLA-F were further identified by SAVEXIS for their specific interaction with VISTA [48]. Based on a high-throughput ECD-interaction screening method called Conditioned Media Alpha Screening, KIR2DL5 (an orphan NK-cell protein) was identified to bind specifically with the poliovirus receptor (PVR) on tumor cells. An anti-KIR2DL5 antibody that effectively blocks the PVR–KIR2DL5 interaction significantly increased the cytotoxicity of LAK effector cells [191]. Analysis of the extracellular interaction network of 445 members of the immunoglobulin superfamily (IgSF) has established a PPI network containing 557 high-confidence interactions, 82% of which are novel discoveries. Approximately 80 protein interactions (~15% of the network) were significantly associated with either an improved or worsened clinical outcome in a large phase 2 clinical trial, suggesting predictive network signatures may aid in the dissection of large patient cohort data [192].

4-1BB is an inducible co-stimulatory receptor expressed on activated T-cells, NK cells, and antigen-presenting cells. Using expression cloning with a cDNA library of EL4 cells, 4-1BBL was identified as the ligand of 4-1BB [193]. Activation of the 4-1BB/4-1BBL pathway triggers immune cell proliferation and activation, particularly of T and NK cells, and agonistic antibodies, urelumab and utomilumab, are in clinical trials [194]. A similar approach was used to identify CD40/CD40L interaction [195, 196], Tim3/galectin-9 interaction [197], and the immunoglobulin-like transcript 4 (ILT-4)/human SEMA4A (hSEMA4A) pathway [198].

HLA-independent unconventional T-cells show universal cytotoxicity against a variety of tumors but do not kill normal tissue cells. Genome-wide CRISPR/Cas9 screening revealed that the monomorphic MHC-1-related protein MR1 is the molecular ligand for the TCR on T-cell clone MC.7.G5. Patient-derived T-cells transfected with this TCR can kill both autologous and nonautologous melanomas, providing a novel target for pan-cancer and pan-population immunotherapy [149]. CRISPR/Cas9 screening in K562 cells, using KIR3DS1-Fc as the bait protein, identified the interaction between KIR3DS1 and heparan sulfate proteoglycans, an interaction that may play a role in NK-cell receptor signaling and target cell recognition [199]. Genome-wide CRISPR screening in macrophages revealed that the G-protein-coupled receptor GPR84 mediates enhanced phagocytosis of APMAP-deficient cancer cells [200].

Secretome interaction arrays identified FGL1 as the major ligand for LAG-3 for T-cell inhibition, and anti-FGL1, or anti-LAG-3 antibodies blocking the FGL1–LAG-3 interaction enhanced the cytotoxicity effect of T-cells [75], with least 13 agents targeting LAG-3 having been subjected to clinical trials [201]. Using a similar approach, a T-cell regulator Siglec-15 was identified [79]. Antibodies blocking Siglec-15 have been shown to inhibit the growth of established tumors in multiple tumor models [202]. On the basis of preclinical functional activity, an anti-Siglec-15 mAb, NC318, is currently being evaluated in phase I/II clinical trials in advanced solid tumors (NCT03665285). B7-H2/CD28 and CTLA-4 [76, 203], B7-H5/CD28H [77], SALM5-HVEM [142], and CD8α-PILRα [204] interactions have also been validated or identified using genome-scale secretome interaction arrays, and the safety and effectiveness of these targets need to be explored in the clinic.

Immunotherapy targets identified using SPPI screening approaches have shown increased clinical benefits for cancer patients [205–209]. Drug blockade of the PD-1/PD-L1 interaction enhances T-cell responses and mediates antitumor activity in multiple cancer types [210]. Seven PD1/PDL1 immune checkpoint antibodies have been approved by the FDA for the treatment of various tumor types [205]. Emerging targets also are undergoing clinical studies for cancer monotherapy or combination therapy [190, 194, 198, 211], such as LAG-3 (IMP321 (Immuntep®), NCT00349934; BMS-986016, NCT01968109), 4-1BB (Urelumab, NCT02253992), and CD47 (Hu5F9-G4, NCT02216409) (Table 3). Immunotherapy also provides a promising approach for the treatment of inflammatory disorders, including atherosclerosis [224], fibrotic diseases [225], systemic lupus erythematosus [226, 227], and pulmonary arterial hypertension [228].

**Table 3.** Examples of immunotherapy targets identified by SPPI screening and related clinical trials.

Screening approaches	PPI	Application	Clinical trials	Ref.
ECD-based high-throughput screening	VISTA(B7-H5)/VSIG-3, PSGL-1	Co-inhibitory receptors	NCT02671955 NCT04475523 NCT05082610	[188–190, 212, 213]
	CD177/PDPN	Co-inhibitory receptors	NA	[214]
	KIR2DL5/PVR	NK-mediated therapy	NCT01248455	[191, 215]
Expression cloning	4-1BB/4-1BBL	Co-stimulatory pathway	NCT02179918 NCT01307267 NCT02444793 NCT02315066	[193, 216]
	CD40/CD40L	Co-stimulatory pathway	NCT01103635 NCT02304393 NCT02482168 NCT03123783	[195, 217]
	OX40/OX40L	Co-stimulatory pathway	NCT01644968 NCT02410512 NCT02315066 NCT02221960	[218, 219]
	ILT-4/SEMA4A	Myeloid immunosuppressive receptors	NCT04669899 NCT03564691	[143]
	CD47/ SIRPa	Myeloid immunosuppressive receptors	NCT02216409 NCT02953509 NCT02890368	[150, 220]
	PD-L1/B7-1	Co-inhibitory receptors	NCT03949231 NCT02840058 NCT05325684 NCT03952065	[221, 222]
CRISPR technology	MR1/MC.7.G5 TCR	TCR-T	NA	[149]
	KIR3DS1/heparan sulfate proteoglycans	NK-mediated therapy	NCT01248455	[199]
Genome-scale secretome interaction arrays	B7-H5/CD28H	Co-inhibitory receptors	NCT02671955	[77]
	Lag-3 /FGL1	Co-inhibitory receptors	NCT00349934 NCT02614833 NCT01968109 NCT02460224	[75, 201, 223]
	Siglec-15/T-cell receptors	Co-inhibitory receptors	NCT03665285	[79, 202]

## Discussion and perspectives

**Figure 8.**
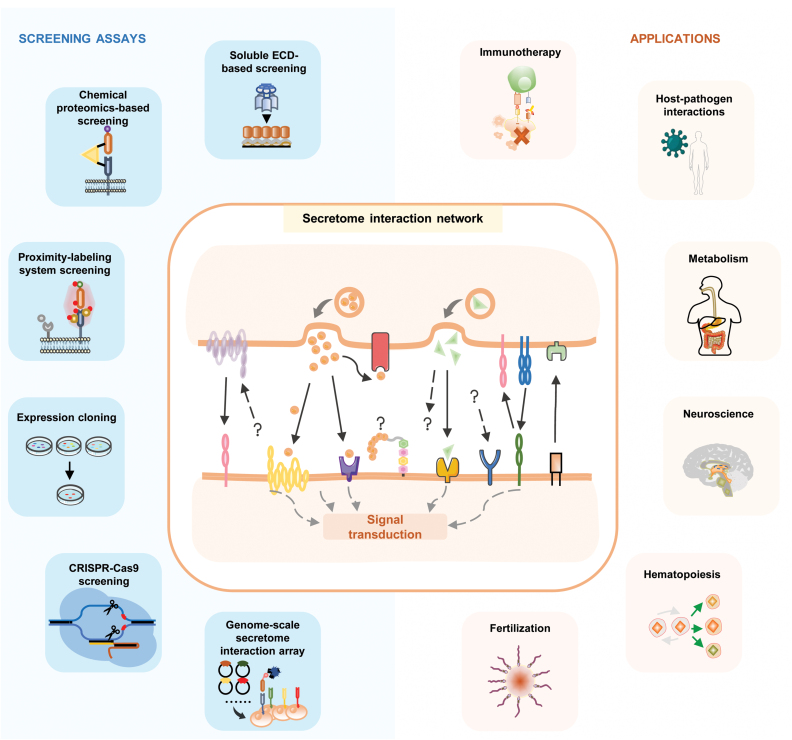
Graphical summary of SPPI screening assays and their applications.

Advances in secretome protein–protein interaction profiling have provided a powerful set of tools to investigate the molecular mechanism underlined a diverse range of physiological and pathological processes (Fig. 8). These approaches have the ability to uncover independent and overlapping SPPIs based, in part, on the biophysical nature of the receptor under investigation. The principles, advantages, and disadvantages of the various SPPI approaches are outlined in Table 1.

Recombinant ECD-based approaches offer a convenient way for the intuitive detection of SPPIs. They can be used to study single-pass transmembrane proteins, GPI-anchored membrane proteins, and secreted proteins. Multi-pass transmembrane proteins such as GPCRs are usually excluded, because of their discontinuous ectodomains. Given the requirement for intensive protein production, it is important to avoid loss of activity of the purified proteins. In addition, as the systems are composed purely of recombinant proteins, it is crucial to validate to what extent the identified interactions are physiologically relevant.

In cell-based SPPI approaches, full-length membrane proteins are expressed either endogenously or ectopically on the cell surface, maintaining protein structure and function and enabling the detection of the various types of SPPIs that are engaged single- or multi-pass membrane proteins. The approach allows analysis of SPPIs under physiological conditions, in cell-based assays, and weaker PPIs are more likely to be detected. Weaknesses of such cell-based screening include the problem that lethal clones will be lost, and membrane proteins with low surface expression are hard to detect.

Library source and size are also major limiting factors for these cell-based approaches. A stimulus-sensitive cell line is usually required to provide a secretome reservoir, and the approach involves expression cloning, proximity labeling or cross-linking, and CRISPR knockout screening, limiting the identified SPPIs to specific cell types or tissues. The libraries used in genome-scale secretome interaction arrays and CRISPRa screening are “artificial,” in the sense that they are either a curated collection of cDNA clones for ectopic expression or involve the activation of endogenous gene expression via a similarly curated sgRNA library. Both could theoretically include most (or even all) secretome proteins. Similar to expression cloning and CRISPR KO screening, CRISPRa screening usually results in the selection of clones with strong phenotypes. In addition, CRISPR “hits” may not necessarily represent direct-binding receptors, but could instead be host factors modulating related biological processes. Genome-scale secretome interaction array analysis involves the individual measurement of each cDNA clone in the library, which, although time and cost consuming, enables generation of a comprehensive picture of the SPPIs for the target at genomic level in one round of screening.

Measurement of the interactions encompassing multi-pass transmembrane protein or heteromeric receptors remains a considerable technical challenge. Most of the current screening approaches require the bait protein to be in soluble form, and are, therefore, suitable for secreted proteins, GPI-anchored proteins, and type I and II single-pass membrane proteins. Multi-pass membrane proteins and membrane protein complexes are hard to meet the criteria of being a soluble bait due to the discontinuous or heteromeric ectodomains. Virus-like particles (VLPs) may provide a strategy to overcome this issue. VLPs are nanoscale structures made up of assembled viral proteins that lack viral genetic material and have been widely used in vaccine development, drug delivery, and cell targeting [229–232]. VLPs can be engineered to display the native target membrane proteins or complex on the recombinant VLP surface, which has been applied to seven-transmembrane GPCRs and single-pass transmembrane proteins, for antibody development [233–235]. VLP technique may allow various types of membrane proteins to function as soluble probes for high-throughput SPPI screening.

A global map of the secretome members is required. Algorithms based on SP and transmembrane segments, such as SignalP, Phobius, and SPOCTOPUS, have been established to predict secreted and transmembrane proteins [236–239]. However, not all secreted proteins adhere to the “classical” SP-dependent pathway, including vital proteins such as IL-1b, FGF1, FGF2, S100A8, and S100A9 [240, 241]. The prediction of non-classical secretion in eukaryotes is challenging despite various methods that have been developed [240, 241]. The annotation of cell surface localization for transmembrane proteins remains incomplete. According to an MS-derived cell surface protein atlas (CSPA), 247 out of 1058 human transmembrane (TM) proteins and 242 out of 938 mouse TM proteins that are identified on cell surfaces lack Uniprot annotations [10]. In additionally, of the identified nonTM proteins residing on the cell membrane, > 90% are non-GPI-anchor proteins [10]. Their authenticity as cell surface proteins and how they associate with cell membranes require plenty of experimental work. The authors further developed a surfaceome predictor, SURFY, using machine learning of the CSPA dataset [11]. Hence, the development of computational and experimental approaches for protein localization is necessary for synergistic cooperation to enhance our understanding of the secretome.

Finally, SPPIs form highly connected and complicated networks for developmental, physiological, and pathological regulation [17, 25, 26]. A comprehensive SPPI database could significantly aid in gene studies and provide a critical dimension of cell–cell communication (CCC) when integrated with spatial and/or single-cell transcriptomics to decipher microenvironment [242–244]. Currently, databases for CCC analysis, such as the commonly used CellphoneDB, encompass ~1900 SPPIs, covering only ~580 secretome proteins [242]. A substantial portion of the SPPI network remains unidentified. High-throughput SPPI screening has facilitated the generation of unprecedented data, enabling the construction of condition-specific SPPI networks and understanding the cell communication with the extracellular environment or response to pathogen invasion. For example, in 2009, a cell surface interaction network of 49 bait- and 52 prey proteins was analyzed to identify 34 novel neural recognition signals in zebrafish [85]. In 2013, focusing on 202 secretome genes in *Drosophila melanogaster*, 106 SPPIs were identified out of 20,503 candidate protein pairs, revealing an extracellular network related to neuronal and developmental functions [45]. In 2020, an ECD-based screening of 564 human immunoglobulin superfamily (IgSF) proteins (564 × 564 binary interactions) was performed, and 426 novel SPPs were identified [26]. In the same year, with a similar approach, the extracellular interaction network among 445 human IgSF members and 1364 human single-pass transmembrane proteins was analyzed, yielding 557 high-confidence SPPIs, with 80 of them associated with either improved or worsened clinical outcomes [192]. These studies pave the way for investigating cell microenvironments or niches in tissues. Nevertheless, huge tasks remain to expand the screening scale through diverse approaches for integration and cross-validation to establish the reliable and comprehensive SPPI atlas.
